# Trehalose Inhibits A53T Mutant α-Synuclein Overexpression and Neurotoxicity in Transduced PC12 Cells

**DOI:** 10.3390/molecules22081293

**Published:** 2017-08-08

**Authors:** Juan Zhao, Xiuling Zhi, Luanfeng Pan, Ping Zhou

**Affiliations:** 1 State Key Laboratory of Molecular Engineering of Polymers, Department of Macromolecular Science, Fudan University, Shanghai 200433, China; 13110440015@fudan.edu.cn; 2 Laboratory of Molecular Biology, Shanghai Medical College, Fudan University, Shanghai 200032, China; zhixiuling@fudan.edu.cn

**Keywords:** α-synuclein, trehalose, transduced PC12 cell, Parkinson’s disease

## Abstract

Fibrillar accumulation of A53T mutant α-synuclein (A53T-AS) in Lewy bodies is a symptom of Parkinsonism. Inhibitions of the overexpression and fibrillar aggregation of α-synuclein (AS) in vivo could be a promising strategy for treating Parkinson’s disease (PD). In this study, at concentrations lower than 1 mM, trehalose decreased the A53T-AS expression level in transduced PC12 cells. Although H_2_O_2_ and aluminum ions increased the expression level and neurotoxicity of A53T-AS in cells, proper trehalose concentrations inhibited the event. These studies adequately prove that trehalose at an appropriate dose would be potentially useful for PD treatment.

## 1. Introduction

Parkinson’s disease (PD) is the second most common disease among nine inherited devastating neurological disorders. Clinically, PD is a slowly progressive movement disorder characterized by tremor, muscle rigidity, imbalance, and bradykinesia. These symptoms are due to the progressive loss of dopaminergic neurons from the substantia nigra, which is a small area in the midbrain. Moreover, some surviving nigral dopaminergic neurons contain cytosolic filamentous inclusions, known as Lewy bodies and Lewy neurites [1].

α-Synuclein (AS) is an intrinsically disordered protein [2,3,4] that localizes within presynaptic terminals in the central nervous system, and is the main component of Lewy bodies [5]. Genetic duplication or triplication of the AS locus causes severe PD [6]. Moreover, mutants of A53T, A30P, E46K, G51D, and H50Q in AS are responsible for familial PD [7,8,9]. Although mutations of AS occur infrequently, they share many of the phenotypic characteristics observed in sporadic PD, and thus could still aid in the revelation of the key biochemical and pathogenic pathways either in genetic or sporadic PD [10]. To date, transgenic mice and flies with the expression of human wild-type or mutant AS exhibited the abnormal cellular accumulation of AS and neuronal dysfunction [11], which implies that AS plays a role in the pathogeneses of both familial and sporadic PD. Moreover, recent reports demonstrated that the intracellular overexpression of AS increased the generation of intracellular reactive oxygen species (ROS), such as H_2_O_2_, caused mitochondrial dysfunction and cell death [12,13], and regulated intracellular transportation, including synaptic vesicle fusion and neurotransmitter release [14].

Currently, PD is considered to be an “environmental” disease [15]. The postmortem analysis of brain tissues from PD patients confirmed considerably increased contents of metal ions, such as iron, zinc, and aluminum ions in the Parkinsonian substantia nigra [16]. Those metal ions have been capable of triggering the structural transformation, β-sheet-rich aggregation, and fibrillation of AS [17]. Among those metals, aluminum ion (Al (III)) is the most efficient element to accelerate the formation of toxic aggregate species of AS [18]. Therefore, inhibition of the overexpression and production of neurotoxic species of AS could be effective treatments for PD.

**Figure 1:**
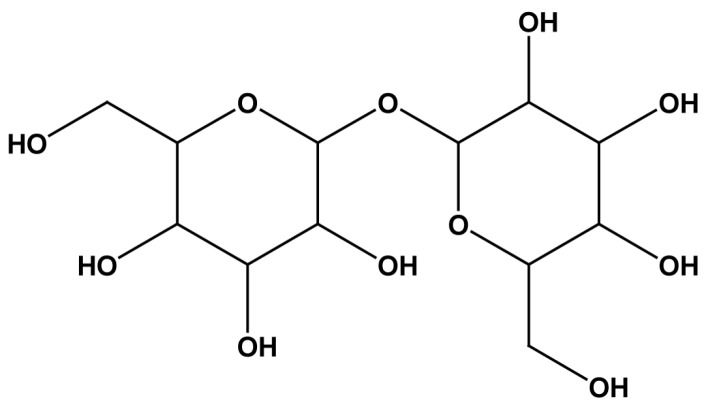
Chemical structure of trehalose.

Trehalose is a non-reduced disaccharide (Figure 1) that has attracted concern recently. In the recent work of Yu et al. [19], at a relatively low concentration, trehalose can inhibit the fibrillation of A53T mutant α-synuclein (A53T-AS) in vitro and even disaggregate the preformed fibrils and protofibrils into the random coil, whereas at higher concentration, trehalose inhibits the formation of fibrils and stabilizes the A53T-AS oligomers. However, on a cellular basis, the neuroprotection mechanism of trehalose still remains unknown, and the manner to control trehalose to an appropriate level for PD treatment would be a challenge before the strategy comes into clinical applications.

The present work aimed to investigate the effect of trehalose at concentrations lower than 10 mM on the overexpression of A53T-AS, and the neuroprotection against the protein aggregation by using PC12 cells as model cells that induce A53T-AS expression. This problem has been receiving limited attention in the past research. We also sought to demonstrate trehalose as a neuroprotective agent for the treatment of PD.

## 2. Results

### 2.1. Inhibitory Effect of Trehalose on the Expression of A53T-AS in the Transduced PC12 Cells

**Figure 2:**
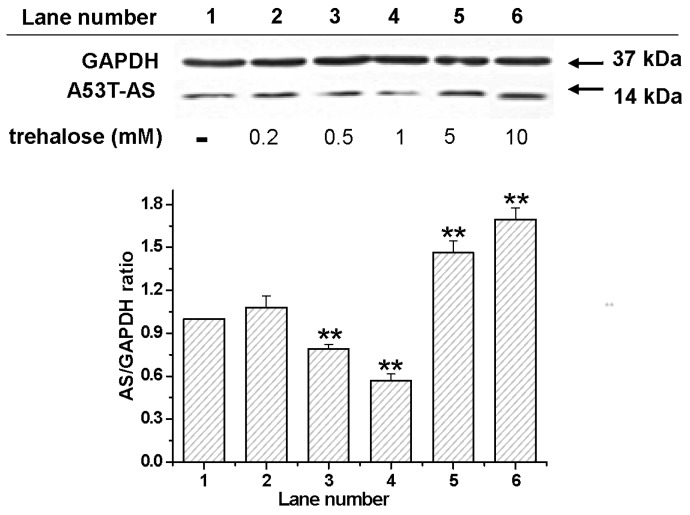
Western blot analyses for A53T mutant α-synuclein (A53T-AS) expression levels in transduced PC12 cells with different concentrations of trehalose. GAPDH (glyceraldehyde-3-phosphate dehydrogenase) lanes were used to calibrate the loaded protein concentration. Error bars = SD, *n* = 3, ** *p* < 0.01 vs. control group.

**Figure 3:**
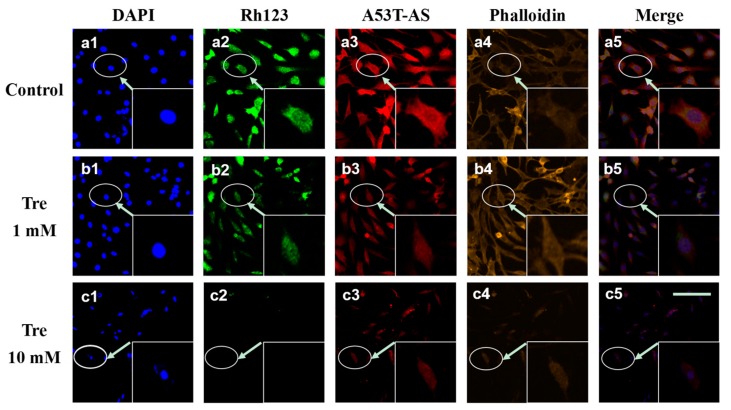
Laser scanning confocal microscope (LSCM) images of a cell nucleus (blue, 4′,6-diamidino-2-phenylindole (DAPI)), mitochondria (green, Rh123), A53T-AS (red, rabbit anti-human AS antibody), actin filament (yellow, phalloidin), and a combination of a cell nucleus, mitochondria, and A53T-AS in transduced PC12 cells treated with 0, 1 and 10 mM trehalose for 48 h. Scale bars = 200 µm. The inserted image is the enlarged version of the cell indicated by an arrow. Every image shown is representative of three repeated experiments.

To reveal the effect of trehalose on A53T-AS overexpression, we employed the rat pheochromocytoma cell line PC12 expressing A53T-AS. Figure 2 shows that, at a concentration lower than 1 mM, trehalose inhibited A53T-AS expression after co-incubation for 48 h, whereas A53T-AS expression was promoted with trehalose at concentrations higher than 1 mM in a dose-dependent manner. Moreover, cytoplasmic inclusions containing A53T-AS (red) and the mitochondrial membrane potential (MMP, green) monitored by the fluorescent dye Rh123 were observed by using LSCM (Figure 3). The Rh123 probe is a cell-permeable cationic dye that preferentially enters into mitochondria based on highly negative MMP. Figure 3 shows that when cells were treated with 1 mM trehalose, A53T-AS expression was slightly inhibited, and the MMP level was not obviously reduced, compared with control cells without trehalose. By contrast, the MMP level was significantly reduced, and cells were obviously damaged, when cells were treated with 10 mM trehalose. The effect of trehalose on the morphology of cells was also observed by labeling actin filament with phalloidin (yellow). After 48 h of treatment, the morphologies of cells treated with 10 mM trehalose were severely damaged and shrank, whereas those treated with 1 mM trehalose displayed a normal spindle-like morphology (Figure 3).

**Figure 4:**
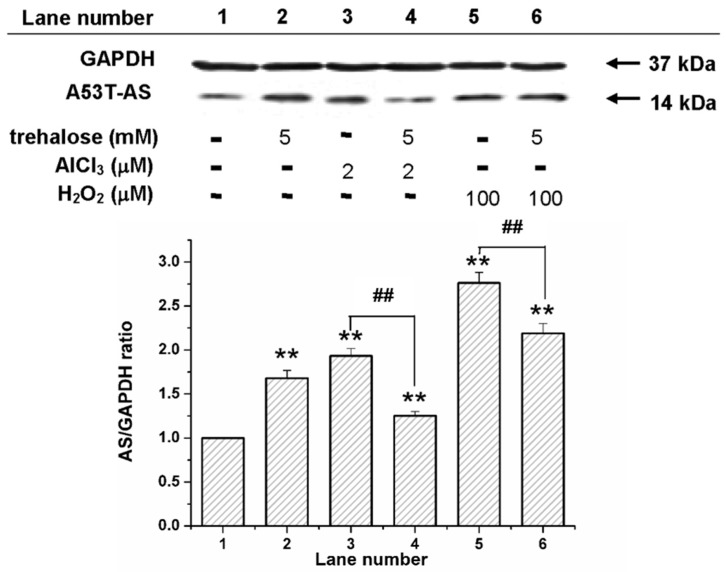
Western blot analyses for A53T-AS expression levels in transduced PC12 cells with and without trehalose, Al (III), and H_2_O_2_. GAPDH (glyceraldehyde-3-phosphate dehydrogenase) lanes were used to calibrate the loaded protein concentration. Error bars = SD, *n* = 3, ** *p* < 0.01 vs. control group; ## *p* < 0.01 vs. 2 µM Al (III) group or 100 µM H_2_O_2_ group.

**Figure 5:**
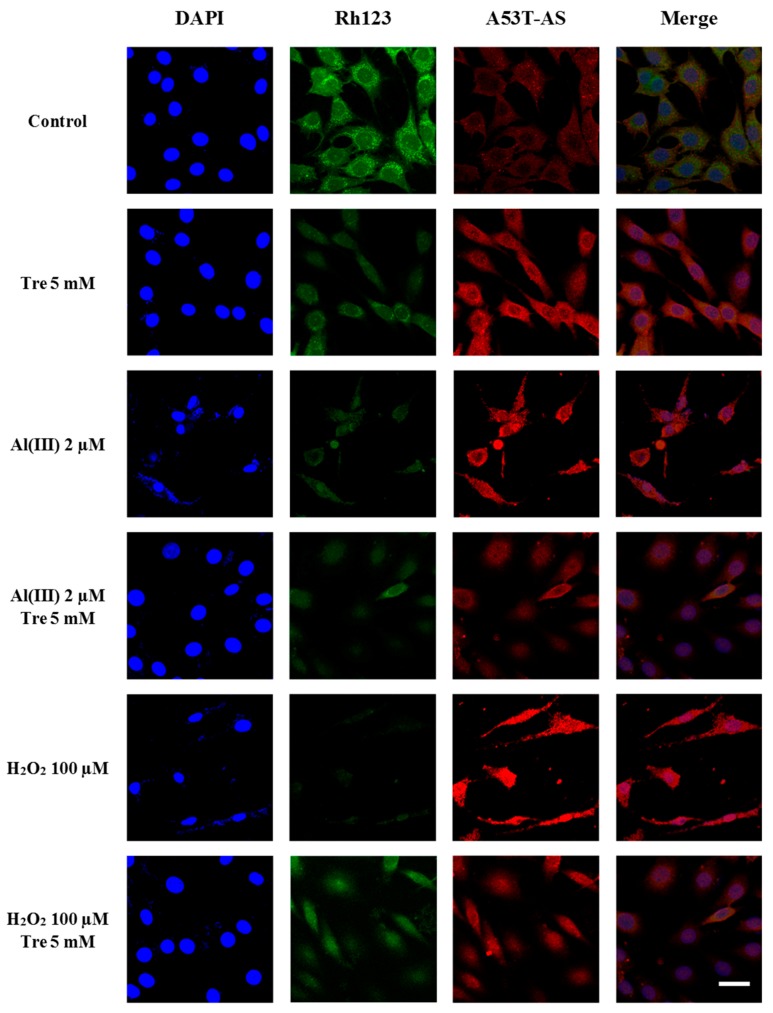
LSCM images of a cell nucleus (blue, DAPI), mitochondria (green, Rh123), A53T-AS (red, rabbit anti-human AS antibody), and a combination of cell nucleus, mitochondria, and A53T-AS in transduced PC12 cells treated with and without 5 mM trehalose, 2 µM Al (III), 10 µM H_2_O_2_ and the combination of H_2_O_2_, aluminum and trehalose for 48 h, respectively. Scale bars = 20 µm. Every image shown is representative of three repeated experiments.

Figure 4 shows that at the co-incubation of transduced PC12 cells with 100 µM H_2_O_2_ and 2 µM Al (III), A53T-AS expression level was significantly higher than that with 5 mM trehalose. To study the effect of trehalose on the Al (III)-induced expression of A53T-AS, transduced PC12 cells were incubated with 2 µM Al (III) along with 5 mM trehalose. Compared with those samples solely containing Al (III) or trehalose, A53T-AS expression was apparently inhibited by Al (III) and trehalose (Figure 4). To further study the effect of trehalose on H_2_O_2_-induced A53T-AS expression, transduced PC12 cells were incubated with 100 µM H_2_O_2_ along with 5 mM trehalose. Compared with those samples containing H_2_O_2_, A53T-AS expression in cells was slightly decreased (Figure 4). To further study the mechanism of trehalose on Al (III)- or H_2_O_2_-induced A53T-AS overexpression in the transduced PC12 cells, we also used LSCM to analyze the distribution of A53T-AS in the cells qualitatively, and Rh123 fluorescence assay to investigate MMP inside the cells. As shown in Figure 5, we found that both 2 µM Al (III) and 100 µM H_2_O_2_ increased the expression of A53T-AS and decreased the MMP. However, when the cells were incubated in the presence of Al (III) or H_2_O_2_ along with 5 mM trehalose, the A53T-AS expression was obviously inhibited, and the MMP level was increased, compared with the sample with Al (III) or H_2_O_2_ alone. 

### 2.2. Effect of Trehalose on the Viability of the Transduced PC12 Cells

**Figure 6:**
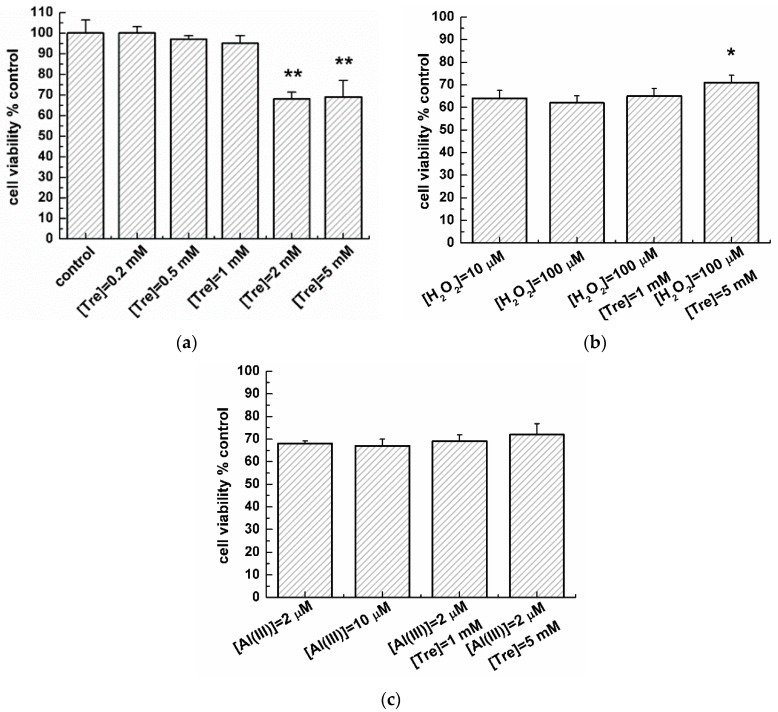
Effect of trehalose on the viability of transduced PC12 cells by MTT assay. (**a**) Comparison of cell viabilities in the absence and presence of various concentrations of trehalose. Error bars = SD, *n* = 5, ** *p* < 0.01 vs. control group; (**b**) Cell viability with H_2_O_2_ or H_2_O_2_/trehalose mixture. Error bars = SD, *n* = 5, * *p* < 0.05 vs. 100 µM H_2_O_2_ group; (**c**) Cell viability in the presence of Al (III) or Al (III)/trehalose mixture.

The effect of trehalose on the viability of transduced PC12 cells with or without H_2_O_2_ and Al (III) was studied by 3-(4,5-dimetylthiazol-2-yl)-2,5-diphenyltetrazolium bromide (MTT) assay (Figure 6). Figure 6a shows that when there was co-incubation of the transduced PC12 cells with trehalose at concentrations lower than 1 mM for 48 h, a slight reduction in the viability of transduced PC12 cells was observed, compared with that of the control group (without trehalose). In contrast, at co-incubation of transduced PC12 cells with trehalose at concentrations higher than 1 mM, the transduced PC12 cell viability was reduced abruptly by 33%. Besides, at concentrations of 10 and 100 µM, H_2_O_2_ significantly attenuated the cell viability to 63% (Figure 6b). Compared with the cell viability of those cells containing only 100 µM H_2_O_2_ or 5 mM trehalose, the cell viability with 5 mM trehalose and 100 µM H_2_O_2_ was obviously increased as co-incubation of transduced PC12 cells with 100 µM H_2_O_2_ and 5 mM trehalose furthered. However, in the presence of 1 mM trehalose with 100 µM H_2_O_2_, the cell viability was not apparently increased. Moreover, at concentrations of 2 and 10 µM, Al (III) significantly attenuated the cell viability to 68% (Figure 6c). Further co-incubation of transduced PC12 cells with 2 µM Al (III) and 5 mM trehalose slightly increased the cell viability, compared with cells that solely contain Al (III) or trehalose. However, the cell viability was not apparently increased with 2 µM Al (III) and 1 mM trehalose.

## 3. Discussion

Understanding the impact mechanism of trehalose on A53T-AS overexpression and neurotoxicity on a cellular basis is a paramount issue for the development of trehalose for clinical applications. AS fibrillation plays an important role in neurodegeneration in Parkinson’s disease; in particular, the oligomeric fibrillary aggregates of AS are toxic to neurons [20]. Combining results presented in this work and from previous work [19], cellular experiments reported here provide an insight into the role of trehalose affecting A53T-AS overexpression and neurotoxicity. Protofibrils and soluble oligomeric aggregates are the primary toxic species, and are responsible for resultant diseases [21]. These protofibrils and oligomeric aggregates were formed during the early stage of protein aggregation, inducing the generation of ROS [21]. As in the present work, H_2_O_2_ is one of the ROS that induced the intracellular overexpression of A53T-AS. Considering the effect of trehalose on the fibrillation of A53T-AS in the previous work [19], trehalose at concentrations lower than 1 mM in PC12 cells could exhibit a similar inhibitory effect on the fibrillation of A53T-AS as that of trehalose at the concentration of approximately 10 mM in the protein system on a molecular basis in vitro [19]. That the trehalose concentration required for the inhibition of A53T-AS fibrillation in cells is much lower than that in protein sample is reasonable, because A53T-AS expression level in transduced PC12 cells is much lower than that of the studied protein sample in vitro. Therefore, low trehalose concentrations decreased the expression level of A53T-AS in transduced PC12 cells. This was possibly due to the inhibition of A53T-AS fibrillation, and even the disaggregation of the preformed amorphous aggregates into the random coil conformer. In contrast, high trehalose concentrations induced the overexpression of A53T-AS, possibly due to the stabilization of A53T-AS oligomers. Moreover, Al (III) efficiently accelerated the formation of A53T-AS toxic species [22], possibly inducing the overexpression of A53T-AS in transduced neuronal cells, which links the correlation between the formation of A53T-AS toxic species and A53T-AS overexpression in transduced PC12 cells.

Sugar transportation onto cells is inhibited by several metals, such as nickel, cobalt, aluminum, and cadmium [23,24]. The inhibition is involved in the interaction of those metals with the polyphosphate membrane, which causes a conformational change of protein at some active sites and hinders the sugar transportation. Although H_2_O_2_ and Al (III) can accelerate the formation of A53T-AS toxic species, and the trehalose concentration (5 mM) itself is also toxic, the complex of Al (III) with trehalose could hinder the toxicity of Al (III) in cells by preventing A53T-AS conformational transition. However, when low trehalose concentrations were co-incubated with Al (III), the effect was not sufficiently strong to ameliorate Al (III) toxicity in cells.

Furthermore, the mutant A53T-AS was highly toxic [25], and cell toxicity was caused directly by A53T-AS expression [13]. The induced A53T-AS expression in transduced PC12 cells could elevate the mitochondrial cytochrome C release and increase the endoplasmic reticulum stress and consecutively produce the excess ROS [13], causing the oxidative stress [26]. Oxidative stress is common in the pathogenesis of age-related neurodegenerative diseases [27]. Two harmful effects emerged from the increased intracellular oxidative stress, as follows: the damage of cellular components, such as DNA and protein, and the activation of specific stress-signaling pathways [28]. These effects considerably led to the death of AS-induced cells [29] and influenced the cellular processes involved in PD [30]. Trehalose is an autophagy enhancer [31] capable of preserving the mitochondrial membrane integrity [32] and preventing behavioral and neurochemical deficits [33]. Mitochondrial damage results from the intracellular ROS production, which causes changes in the mitochondrial membrane potential during apoptosis [34]. In the present work, trehalose affected the mitochondrial morphology of cells. Low trehalose concentrations did not change the mitochondrial morphology; however, high trehalose concentrations disrupted the mitochondrial morphology, which decreased the MMP of cells. Therefore, we showed in Figure 6 that low trehalose concentrations increase the cell viability possibly by inhibiting the intracellular A53T-AS overexpression. In contrast, high trehalose concentrations attenuate the cell viability by increasing the intracellular A53T-AS overexpression. Lan et al. also demonstrated that high trehalose concentrations reduced cell viability [35]. They treated cells with 10~50 mM trehalose for 24 h, while we used relatively low concentrations (0.2~5 mM) incubated with cells for 48 h to investigate the effect of trehalose on A53T-AS transduced PC12 cells for an extended period of time. Although the used concentrations of trehalose were different, they have the same impact trend. Lan et al. suggested that trehalose promoted the clearance of A53T-AS, which was mediated through the macroautophagy pathway [31,35]. It seems reasonable to speculate that this small molecule inhibits the overexpression of A53T-AS through a variety of different mechanisms. Furthermore, proper trehalose concentrations decrease the Al (III)- or H_2_O_2_-induced cell death by inhibiting the Al (III)- or H_2_O_2_-induced intracellular A53T-AS overexpression, and decreasing ROS in cells (Figure 5).

**Figure 7:**
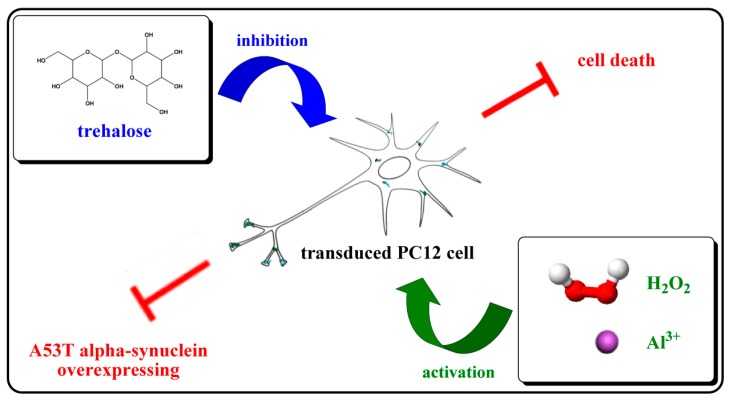
Scheme illustrating the effect of trehalose on the in A53T-AS transduced PC12 cells. H_2_O_2_ and Al (III) increased the expression and neurotoxicity of A53T-AS in the cells, while trehalose at proper concentrations inhibited the events.

Proper trehalose concentrations could inhibit the intracellular A53T-AS overexpression induced by Al (III) and H_2_O_2_, decrease the neurotoxicity of A53T, and protect the integrity of cells against the oxidative damage (Figure 7). Thus, trehalose could be potentially used for PD treatment. As high trehalose concentrations are cytotoxic, the usage and dosage should be in accordance with the physical condition of patients’ conditions in future applications. In order to achieve the maximum effect of trehalose as a neuroprotective agent for human use, strategies of shunning the drawbacks of high dosages are necessary. Controlled-release systems can critically improve its bioavailability, and weaken the cytotoxicity associated with high dosages, which is typically required for optimum response with a drug. Certainly, studying more intracellular pathways is necessary to elucidate the mechanism underlying trehalose inhibition of Al (III)- or H_2_O_2_-induced cell death.

## 4. Materials and Methods

### 4.1. Generation of Constructs and Transduction of PC12 Cells

The PC12 cell is a cell line derived from a pheochromocytoma of the rat adrenal medulla and was purchased from Shanghai Institutes for Biological Sciences, CAS, Shanghai, China. PC12 cells that can overexpress the wild-type AS were prepared as described in our previous reports [36]. Briefly, PCR (Applied Biosystems, Waltham, MA, USA) was used to amplify the Open Reading Frame of human A53T-AS. PCR product was then cloned into a pDrive vector (Invitrogen). A53T-AS in pcDNA3.1 was subcloned in PatI-XhoI sites of a pLenti6/V5 expression vector (Invitrogen; Carlsbad, CA, USA) at the downstream of a cytomegalovirus promoter. The orientation and sequence of the construct were confirmed by restriction analysis and DNA sequencing. Then, lentiviral stocks produced by co-transfecting the optimized packaging plasmid mixture, and pLenti expressing construct into the 293T cell line, were produced according to the manufacturer’s protocols (The ViraPower^TM^ Lentiviral Expression System, Invitrogen), as previously described [37]. After A53T-AS in lentiviral stocks had been transduced into the PC12 cell line, the individual stably transduced colony was subsequently selected using blasticidin (Invitrogen). Finally, A53T-AS expression level was assessed by Western blot.

### 4.2. Cells Expressing A53T-AS Analyzed by Western Blot

The transduced PC12 cells expressing A53T-AS were grown in Dulbecco’s modified Eagle’s medium (DMEM) containing 6.7% horse serum, 3.3% fetal bovine serum (FBS), 1% l-glutamine (3.6 mM), and 1% penicillin/streptomycin antibiotics under 5% CO_2_ atmosphere at 37 °C. Cells were harvested from flasks and plated in six-well polystyrene plates (Corning Inc., New York, NY, USA) with 5 × 10^5^ cells in 2.5 mL medium per well, and then incubated at 37 °C for 24 h to attach the plate. Two µM Al (III), 100 µM H_2_O_2_, and 0.2, 0.5, 1, 5 and 10 mM trehalose were then added individually to those samples. The selection of concentrations used for trehalose and H_2_O_2_ was based on our previous study [18]. The same volume of medium was added to control cultures (only PC12 cells present). Cells were then incubated further for 48 h at 37 °C. At the end of incubation, A53T-AS expression in cells was analyzed by Western blot method. Cells were lysed on ice in the lysis buffer with 1% Triton X-100, 50 mM Tris (pH 7.4), 150 mM NaCl, 20 mM iodoacetamide, 1 mM PMSF, and 1% aprotinin for 30 min. After centrifugation at 12,000 *g* for 10 min, supernatants were collected. Protein concentrations in supernatants were determined by BCA method (Bio-Rad, Hercules, CA, USA). Before electrophoresis, cell lysates in supernatants were further diluted with SDS loading buffer (2.5% SDS, 0.38 M Tris, pH 6.8, 20% glycerol, and 0.1% bromophenol blue) and heated at 100 °C for 5 min. A total of 30 μg of lysate was separated by SDS-PAGE, and transferred to polyvinylidene difluoride (PVDF) membranes (Millipore, Billerica, MA, USA). The transformation to PVDF membrane was then blocked in TBST (10 mM Tris-HCl, pH 7.4, 150 mM NaCl, 0.1% Tween-20, 5% non-fat milk) and probed by the rabbit anti-human AS primary antibody (1:1000, CST, Boston, MA, USA). The protein was detected using enhanced chemiluminescence reagents (ECL, Pierce, Waltham, MA, USA), according to the manufacturer’s instruction. The intensity of each band was estimated by densitometric quantification using the Image Lab version 5.2.1 software (Bio-Rad). Data were collected from three independent experiments.

### 4.3. Immunofluorescence Microscopy

The transduced PC12 cells expressing A53T-AS were grown in Dulbecco’s modified Eagle’s medium (DMEM) containing 6.7% horse serum, 3.3% fetal bovine serum (FBS), 1% l-glutamine (3.6 mM), and 1% penicillin/streptomycin antibiotics under 5% CO_2_ atmosphere at 37 °C. Cells were harvested from flasks and seeded on 0.01% poly-l-lysine-coated (Songon Biotech, Shanghai, China) glass slides in 12-well polystyrene plates (Corning Inc., New York, NY, USA), with 5 × 10^4^ cells in 1 mL medium per well, and incubated at 37 °C for 24 h to attach the plate. Then, the pre-incubated medium in each well was aspirated carefully, and 1 mL fresh medium (DMEM containing 1% horse serum, 0.5% FBS, 1% l-glutamine (3.6 mM), and 1% penicillin/streptomycin antibiotics) with 0, 1, and 10 mM trehalose were added individually to each cell well. Cells were incubated for an additional 48 h at 37 °C, and then stained with 10 µM rhodamine 123 (Rh123) (Dojindo Laboratories, Kumamoto-ken, Japan) for 30 min. Cells were fixed in 4% (*w*/*v*) paraformaldehyde in phosphate buffer saline (PBS) for 20 min. Upon washing with PBS, cells were permeated with 0.1% Triton X-100 in PBS and blocked with 3% goat serum (Songon Biotech) in PBS for 30 min at room temperature, subsequently incubated with rabbit anti-human AS primary antibody (1:250, CST, Boston, MA, USA) for 1 h, and then incubated with a secondary antibody (1:250, goat anti-rabbit IgG conjugated to Alexa Fluor 647, CST, Boston, MA, USA) for 1 h. DAPI (4′,6-diamidino-2-phenylindole, Songon Biotech) and TRITC-conjugated phalloidin (Yeasen, Shanghai, China) was used to label nuclei and actin filament, respectively. After a final washing with PBS, coverslips were mounted in the antifade mounting medium (Beyotime, Shanghai, China) and a laser scanning confocal microscope (LSCM, C2^+^, Nikon, Tokyo, Japan) was applied to capture images.

### 4.4. Cell Viability Analysis by MTT Method

Transduced PC12 cells were grown in DMEM containing 6.7% horse serum, 3.3% FBS, 1% l-glutamine (3.6 mM), and 1% penicillin/streptomycin antibiotics under 5% CO_2_ atmosphere at 37 °C. Cells were harvested from flasks and plated in 96-well polystyrene plates with 5.5 × 10^3^ cells in 100 µL medium per well. Cells were incubated at 37 °C for 24 h to attach the plates. Then, the pre-incubated medium in each well was aspirated carefully, and 100 µL fresh medium (DMEM containing 1% horse serum, 0.5% FBS, 1% l-glutamine (3.6 mM), and 1% penicillin/streptomycin antibiotics) with 0.2, 0.5, 1, 2 and 5 mM trehalose or 10 and 100 µM H_2_O_2_ or 2 and 10 µM Al (III) or their mixtures were added individually to each cell well. The same volume of medium was added to the control well (only PC12 cells present). Cells were then incubated for additional 48 h at 37 °C. At the end of incubation, 20 µL fresh DMEM with 3.3 mg/mL thiazolyl blue tetrazolium bromide was added to each well and incubated for additional 4 h at 37 °C for the formation of formazan crystals. The formed crystals were then dissolved by adding 100 µL DMSO in each well and shaking for 20 min at room temperature. The absorbance was measured at 490 nm by Bio-Rad multi-well assay plate reader, and averaged by five replicated wells for each sample and control. The cell viability was calculated by the ratio of the absorbance of studied groups to that of the control group. Data were collected from three independent experiments. Standard deviations were analyzed by one-way ANOVA packaged in Origin 7.5 software (OriginLab, Northampton, MA, USA).

## Abbreviations

PD	Parkinson’s disease
Al (III)	aluminum ion
A53T-AS	A53T mutant α-synuclein
AS	α-synuclein
ROS	reactive oxygen species

## References

[1] Forno L.S. (1996). Neuropathology of parkinson’s disease. J. Neuropathol. Exp. Neurol..

[2] Orr A.A., Wordehoff M.M., Hoyer W., Tamamis P. (2016). Uncovering the binding and specificity of beta-wrapins for amyloid-beta and alpha-synuclein. J. Phys. Chem. B.

[3] Bartels T., Choi J.G., Selkoe D.J. (2011). Alpha-synuclein occurs physiologically as a helically folded tetramer that resists aggregation. Nature.

[4] Dettmer U., Newman A.J., Luth E.S., Bartels T., Selkoe D. (2013). In vivo cross-linking reveals principally oligomeric forms of alpha-synuclein and beta-synuclein in neurons and non-neural cells. J. Biol. Chem..

[5] Siderowf A., Stern M. (2003). Update on parkinson disease. Ann. Intern. Med..

[6] Singleton A.B., Farrer M., Johnson J., Singleton A., Hague S., Kachergus J., Hulihan M., Peuralinna T., Dutra A., Nussbaum R. (2003). Alpha-synuclein locus triplication causes parkinson’s disease. Science.

[7] Zarranz J.J., Alegre J., Gomez-Esteban J.C., Lezcano E., Ros R., Ampuero I., Vidal L., Hoenicka J., Rodriguez O., Atares B. (2004). The new mutation, E46K, of alpha-synuclein causes parkinson and lewy body dementia. Ann. Neurol..

[8] Fares M.B., Ait-Bouziad N., Dikiy I., Mbefo M.K., Jovicic A., Kiely A., Holton J.L., Lee S.J., Gitler A.D., Eliezer D. (2014). The novel parkinson’s disease linked mutation G51D attenuates in vitro aggregation and membrane binding of alpha-synuclein, and enhances its secretion and nuclear localization in cells. Hum. Mol. Genet..

[9] Rutherford N.J., Moore B.D., Golde T.E., Giasson B.I. (2014). Divergent effects of the H50Q and G51D snca mutations on the aggregation of alpha-synuclein. J. Neurochem..

[10] Brice A. (2006). What can we learn from genes responsible for familial forms of parkinson’s disease. Bull. Acad. Natl. Med..

[11] Lee M.K., Stirling W., Xu Y.Q., Xu X.Y., Qui D., Mandir A.S., Dawson T.M., Copeland N.G., Jenkins N.A., Price D.L. (2002). Human alpha-synuclein-harboring familial parkinson’s disease-linked ala-53 -> thr mutation causes neurodegenerative disease with alpha-synuclein aggregation in transgenic mice. Proc. Natl. Acad. Sci. USA.

[12] Tabrizi S.J., Orth M., Wilkinson J.M., Taanman J.W., Warner T.T., Cooper J.M., Schapira A.H.V. (2000). Expression of mutant alpha-synuclein causes increased susceptibility to dopamine toxicity. Hum. Mol. Genet..

[13] Smith W.W., Jiang H.B., Pei Z., Tanaka Y., Morita H., Sawa A., Dawson V.L., Dawson T.M., Ross C.A. (2005). Endoplasmic reticulum stress and mitochondrial cell death pathways mediate A53T mutant alpha-synuclein-induced toxicity. Hum. Mol. Genet..

[14] Darios F., Ruiperez V., Lopez I., Villanueva J., Gutierrez L.M., Davletov B. (2010). α-Synuclein sequesters arachidonic acid to modulate snare-mediated exocytosis. Embo Rep..

[15] Jomova K., Vondrakova D., Lawson M., Valko M. (2010). Metals, oxidative stress and neurodegenerative disorders. Mol. Cell. Biochem..

[16] Dexter D.T., Carayon A., Javoyagid F., Agid Y., Wells F.R., Daniel S.E., Lees A.J., Jenner P., Marsden C.D. (1991). Alterations in the levels of iron, ferritin and other trace-metals in parkinsons-disease and other neurodegenerative diseases affecting the basal ganglia. Brain.

[17] Uversky V.N., Li J., Fink A.L. (2001). Metal-triggered structural transformations, aggregation, and fibrillation of human alpha-synuclein—A possible molecular link between parkinson’s disease and heavy metal exposure. J. Biol. Chem..

[18] Zatta P., Ricchelli F., Drago D., Filippi B., Tognon G. (2005). Aluminum-triggered structural modifications and aggregation of beta-amyloids. Cell. Mol. Life Sci..

[19] Yu W.B., Jiang T., Lan D.M., Lu J.H., Yue Z.Y., Wang J., Zhou P. (2012). Trehalose inhibits fibrillation of A53T mutant alpha-synuclein and disaggregates existing fibrils. Arch. Biochem. Biophys..

[20] Kalia L.V., Kalia S.K., Mclean P.J., Lozano A.M., Lang A.E. (2013). α-Synuclein oligomers and clinical implications for parkinson disease. Ann. Neurol..

[21] Conway K.A., Lee S.J., Rochet J.C., Ding T.T., Williamson R.E., Lansbury P.T. (2000). Acceleration of oligomerization, not fibrillization, is a shared property of both alpha-synuclein mutations linked to early-onset PARKINSON’S disease: Implications for pathogenesis and therapy. Proc. Natl. Acad. Sci. USA.

[22] Exley C. (2006). Aluminium and iron, but neither copper nor zinc, are key to the precipitation of beta-sheets of A beta(42) in senile plaque cores in alzheimer's disease. J. Alzheimers Dis..

[23] Van Steveninck J. (1966). The influence of nickelous ions on carbohydrate transport in yeast cells. Biochim. Biophys. Acta. Biophys. Incl. Photosynth..

[24] Nakamura H. (1964). Adaptation of yeast to cadmium. Jpn. J. Genet..

[25] Tanaka Y., Engelender S., Igarashi S., Rao R.K., Wanner T., Tanzi R.E., Sawa A., Dawson V.L., Dawson T.M., Ross C.A. (2001). Inducible expression of mutant alpha-synuclein decreases proteasome activity and increases sensitivity to mitochondria-dependent apoptosis. Hum. Mol. Genet..

[26] Turnbull S., Tabner B.J., El-Agnaf O.M.A., Moore S., Davies Y., Allsop D. (2001). Alpha-synuclein implicated in parkinson’s disease catalyses the formation of hydrogen peroxide in vitro. Free Radic. Biol. Med..

[27] Paik S.R., Shin H.J., Lee J.H. (2000). Metal-catalyzed oxidation of alpha-synuclein in the presence of copper(ii) and hydrogen peroxide. Arch. Biochem. Biophys..

[28] Souza J.M., Giasson B.I., Chen Q.P., Lee V.M.Y., Ischiropoulos H. (2000). Dityrosine cross-linking promotes formation of stable alpha-synuclein polymers—Implication of nitrative and oxidative stress in the pathogenesis of neurodegenerative synucleinopathies. J. Biol. Chem..

[29] Jiang H.B., Wu Y.C., Nakamura M., Liang Y.D., Tanaka Y.J., Holmes S., Dawson V.L., Dawson T.M., Ross C.A., Smith W.W. (2007). Parkinson’s disease genetic mutations increase cell susceptibility to stress: Mutant alpha-synuclein enhances H_2_O_2_- and sin-1-induced cell death. Neurobiol. Aging.

[30] Finkel T., Holbrook N.J. (2000). Oxidants, oxidative stress and the biology of ageing. Nature.

[31] Lan D., Wang W., Zhuang J., Zhao Z. (2015). Proteasome inhibitor-induced autophagy in PC12 cells overexpressing A53T mutant alpha-synuclein. Mol. Med. Rep..

[32] Casarejos M.J., Perucho J., Lopez-Sendon J.L., Garcia de Yebenes J., Bettencourt C., Gomez A., Ruiz C., Heutink P., Rizzu P., Mena M.A. (2014). Trehalose improves human fibroblast deficits in a new chip-mutation related ataxia. PLoS ONE.

[33] He Q., Koprich J.B., Wang Y., Yu W.B., Xiao B.G., Brotchie J.M., Wang J. (2016). Treatment with trehalose prevents behavioral and neurochemical deficits produced in an AAV alpha-synuclein rat model of parkinson’s disease. Mol. Neurobiol..

[34] Trancikova A., Tsika E., Moore D.J. (2012). Mitochondrial dysfunction in genetic animal models of parkinson’s disease. Antioxid. Redox Signal..

[35] Lan D.M., Liu F.T., Zhao J., Chen Y., Wu J.J., Ding Z.T., Yue Z.Y., Ren H.M., Jiang Y.P., Wang J. (2012). Effect of trehalose on PC12 cells overexpressing wild-type or A53T mutant alpha-synuclein. Neurochem. Res..

[36] Neystat M., Lynch T., Przedborski S., Kholodilov N., Rzhetskaya M., Burke R.E. (1999). Alpha-synuclein expression in substantia nigra and cortex in parkinson’s disease. Mov. Disord..

[37] Wu J.J., Yu W.B., Chen Y., Su Y.R., Ding Z.T., Ren H.M., Jiang Y.P., Wang J. (2010). Intrastriatal transplantation of gdnf-engineered bmscs and its neuroprotection in lactacystin-induced parkinsonian rat model. Neurochem. Res..

